# Exosome-Mediated miR-21 Was Involved in the Promotion of Structural and Functional Recovery Effect Produced by Electroacupuncture in Sciatic Nerve Injury

**DOI:** 10.1155/2022/7530102

**Published:** 2022-01-29

**Authors:** Yu-pu Liu, Yi-duo Yang, Fang-fang Mou, Jing Zhu, Han Li, Tian-tian Zhao, Yue Zhao, Shui-jin Shao, Guo-hong Cui, Hai-dong Guo

**Affiliations:** ^1^Department of Anatomy, School of Basic Medicine, Shanghai University of Traditional Chinese Medicine, Shanghai 201203, China; ^2^Department of Orthopaedics, Shanghai Key Laboratory for Prevention and Treatment of Bone and Joint Diseases, Shanghai Institute of Traumatology and Orthopaedics, Ruijin Hospital, Shanghai Jiao Tong University School of Medicine, Shanghai 200025, China; ^3^Department of Neurology, Shanghai No. 9 People's Hospital, Shanghai Jiaotong University School of Medicine, Shanghai 200011, China; ^4^Academy of Integrative Medicine, Shanghai University of Traditional Chinese Medicine, Shanghai 201203, China

## Abstract

**Purpose:**

Our study is aimed at investigating the mechanism by which electroacupuncture (EA) promoted nerve regeneration by regulating the release of exosomes and exosome-mediated miRNA-21 (miR-21) transmission. Furthermore, the effects of Schwann cells- (SC-) derived exosomes on the overexpression of miR-21 for the treatment of PNI were investigated.

**Methods:**

A sciatic nerve injury model of rat was constructed, and the expression of miR-21 in serum exosomes and damaged local nerves was detected using RT-qPCR after EA treatment. The exosomes were identified under a transmission electron microscope and using western blotting analysis. Then, the exosome release inhibitor, GW4869, and the miR-21-5p-sponge used for the knockdown of miR-21 were used to clarify the effects of exosomal miR-21 on nerve regeneration promoted by EA. The nerve conduction velocity recovery rate, sciatic nerve function index, and wet weight ratio of gastrocnemius muscle were determined to evaluate sciatic nerve function recovery. SC proliferation and the level of neurotrophic factors were assessed using immunofluorescence staining, and the expression levels of SPRY2 and miR-21 were detected using RT-qPCR analysis. Subsequently, the transmission of exosomal miR-21 from SC to the axon was verified *in vitro*. Finally, the exosomes derived from the SC infected with the miR-21 overexpression lentivirus were collected and used to treat the rat SNI model to explore the therapeutic role of SC-derived exosomes overexpressing miR-21.

**Results:**

We found that EA inhibited the release of serum exosomal miR-21 in a PNI model of rats during the early stage of PNI, while it promoted its release during later stages. EA enhanced the accumulation of miR-21 in the injured nerve and effectively promoted the recovery of nerve function after PNI. The treatment effect of EA was attenuated when the release of circulating exosomes was inhibited or when miR-21 was downregulated in local injury tissue via the miR-21-5p-sponge. Normal exosomes secreted by SC exhibited the ability to promote the recovery of nerve function, while the overexpression of miR-21 enhanced the effects of the exosomes. In addition, exosomal miR-21 secreted by SC could promote neurite outgrowth *in vitro*.

**Conclusion:**

Our results demonstrated the mechanism of EA on PNI from the perspective of exosome-mediated miR-21 transport and provided a theoretical basis for the use of exosomal miR-21 as a novel strategy for the treatment of PNI.

## 1. Introduction

Peripheral nerve injury (PNI) is a common clinical disease [1] that is usually caused by trauma, compression, ischemic, and metabolic disorders [2], leading to the loss of sensory and motor neural functions [3–5]. The regeneration of the injured nerve is usually slow and incomplete, while patients suffer from pain and from a lower quality of life after PNI [6]. As a refractory disease, patients with PNI suffer with impairment of function even after the surgical treatment [7–11], for example, 40% of motor function was recovered by autologous nerve transplantation [12]. Thus, researchers have been trying to develop nonsurgical methods for the management of PNI. Electroacupuncture (EA) is defined as a combination of electrical stimulation with acupuncture needles and is capable of promoting the regeneration of the transected median nerve in rats [13] and increasing neuronal stem cell growth [14]. We have confirmed that EA could promote Schwann cells (SC) proliferation and the release of neurotrophic factors (NTFs), including brain-derived neurotrophic factor (BDNF) [15] and nerve growth factor (NGF) [16] after PNI.

miRNAs are involved in a wide range of biological processes [17–20] through base pairing with complementary sequences within mRNA [21]. miRNAs have been regarded as important diagnostic and therapeutic targets [22], and they can regulate many target genes [23–25]. Moreover, EA provides a multichannel, multitarget, and multilink method of treatment [26–30]. Therefore, we aimed to elucidate new mechanisms of EA treatment for PNI via the regulation of miRNAs. It has been proven that the expressions of many different miRNAs are changed after PNI [15, 31–34], indicating its association with the occurrence and development of the nervous system [35, 36]. miRNA-21 (miR-21) expression was upregulated following PNI in the serum [37], local injury tissue [38], and dorsal root ganglion (DRG) [39]. Moreover, miR-21 is strongly associated with nerve repair [40], neuron survival [41], and neuropathic pain [42]. It has been reported that miR-21 can promote the proliferation and survival of SC [43]. As a well-known key player involved in PNI repair, SC is responsible for the formation of the myelin sheath that protects axons and material exchange with the axons [44].

As an excellent drug carrier and new diagnostic marker and therapeutic agent [45–47], exosomes play a significant role in the myelination and nerve regeneration after PNI by transferring proteins or nucleic acids [48, 49]. Current research indicates that exosome-derived stem cells may cause neuroprotective effects, remyelination, and recovery of function after PNI [50]. EA has been found to increase the abundance of exosomes and regulate miRNA and circular RNA in exosomes found in blood circulation [51, 52]. Therefore, a PNI model of rats was constructed to investigate the mechanism by which EA promotes the structural and functional recovery through the delivery of exosome-mediated miR-21, which provides a promising strategy for the treatment of PNI.

## 2. Materials and Methods

### 2.1. Animal Model and Tissue Preparation

SPF-grade male Wistar rats (body weight 260 ± 20 g) were provided by the Animal Experimental Center of Shanghai University of Traditional Chinese Medicine.

#### 2.1.1. Method 1

After a week of adaptive feeding, the rats were anesthetized using 1% pentobarbital (45 mg/kg) and randomly divided into 3 groups (*n* = 10 in each group). For the sham group (Sham), the right sciatic nerve was exposed, and the skin was sutured with the nerve left intact. For the model group (Model), the right sciatic nerve was exposed, and the adventitial layer was sutured after transection injury, followed by skin suture. For the electroacupuncture group (EA), acupuncture needles were inserted into the acupoints, “Huantiao” (GB30) and “Zusanli” (ST36), and connected to positive and negative poles, respectively. Thereafter, the rats were treated using EA after modeling for 7 days (intermittent wave, 20 min each day at an electric current intensity that caused a slight muscle twitch was selected). After treatment, blood was collected from the abdominal aorta and centrifuged at 3000 g at 4°C for 15 min to isolate serum. Total RNA from injured right sciatic nerve was extracted using NucleoZOL (MN, Düren, Germany).

#### 2.1.2. Method 2

After the rats were anesthetized, as described above, the distal segment of the right sciatic nerve (intact or injured) was exposed and transfected with 25 *μ*l of pCT-CD63-GFP plasmid-containing the lentivirus (OBiO Technology, Shanghai, China) and 25 *μ*l of Matrigel (BD Biosciences, Bedford, MA), and then, the skin was sutured. At 7 days after transfection, cross-sections of the right sciatic nerve (10 *μ*m) were prepared after being fixed with 4% paraformaldehyde and embedded with OCT (Sakura Finetek, Tokyo, Japan).

#### 2.1.3. Method 3

After the model was created, as described in Method 1, the rats were randomly divided into 4 groups (*n* = 10 in each group). For the model group (Model), the distal segment of the injured nerve was injected with 25 *μ*l of Matrigel and 25 *μ*l of the lentivirus produced using an empty plasmid (OBiO Technology). The rats also received an injection of 3.75% dimethylsulphoxide (DMSO; Sigma) diluted in saline every 2 days for 3 weeks. For the electroacupuncture group (EA), the lentivirus was injected as the model group, and EA was performed with 1 day rest every 7 days for 3 weeks (same parameter as Method 1). Similar to the model group, these rats also received an injection of DMSO. For the electroacupuncture + GW4869 group (EG), the lentivirus was injected, like the model group, and EA was performed with 1 day rest every 7 days for 4 weeks (same parameters as Method 1), and the rats received an injection of 1 mg/kg GW4869 dissolved in 3.75% DMSO diluted with saline every 2 days for 3 weeks. For the electroacupuncture + miR-21-5p-sponge group (ES), the distal segment of the injured nerve was injected with 25 *μ*l of Matrigel and 25 *μ*l of the lentivirus produced using the miR-21-5p-sponge expression plasmid (OBiO Technology, Shanghai, China). The rats received a DMSO injection, like the model group.

At 3 weeks after the treatment, total RNA of the proximal injured nerve was extracted using NucleoZOL. Cross-sections of the distal right sciatic nerve (10 *μ*m) were prepared after being fixed using 4% paraformaldehyde and embedded in OCT.

#### 2.1.4. Method 4

After modeling as mentioned in Method 1, the rats were randomly divided into 3 groups (*n* = 10 in each group). For the model group (Model), the distal segment of the injured nerve was injected with 25 *μ*l of Matrigel and 25 *μ*l of PBS. For the SC-derived exosomes group (EXO-MC), the distal segment of the injured nerve was injected with 25 *μ*l of Matrigel and 25 *μ*l of SC-derived exosomes (15 *μ*g). For the SC-derived exosomes with the overexpression of miR-21 (EXO-miR-21), the distal segment of the injured nerve was injected with 25 *μ*l of Matrigel and 25 *μ*l of SC-derived exosomes with the overexpression of miR-21 (15 *μ*g). At 3 weeks after treatment, total RNA of the proximal injured nerve was extracted, and 10 mm thick frozen slices of the distal injured nerve were prepared.

### 2.2. Detection of Sciatic Nerve Function Recovery Index

The general condition of the rats was evaluated every week: wounds, skin on the feet, and the activities of the right hind limb muscles and joints were recorded. Three different methods were used to determine the sciatic nerve function recovery index before the rats were sacrificed.

#### 2.2.1. Method 1

For nerve conduction velocity recovery rate (NCV), the NCV of the bilateral sciatic nerve was assessed, as previously described [15]. In brief, the stimulating electrode and the receiving electrode were connected to the proximal and distal ends of the sciatic nerve, respectively, using the copper handle of the acupuncture needle. A RM6240 Biological Signal Collecting System (Chengdu Instrument Factory, Chengdu, China) was used to trigger an electrical stimulation and record compound motor action potentials (CMAPs), while nerve conduction velocity was measured based on the distance between electrodes and the time required for electrical stimulation delivery. The recovery of NCV was subsequently calculated through the ipsilateral/contralateral ratio.

#### 2.2.2. Method 2

For sciatic nerve function index (SFI), the rats were allowed to freely walk on white paper after their hind paws were dipped in ink. PL indicates the length from the heel to the toe, and TS indicates the toe-spread between toes 1 and 5, while IT indicates the toe-spread between toes 2 and 4 (N: normal side; E: experimental side). SFI was calculated as follow: SFI = −38.3 × (EPL − NPL)/NPL + 109.5 × (ETS − NTS)/NTS + 13.3 × (EIT − NIT)/NIT − 8.8. A value closer to 0 indicated better recovery of neurological function.

#### 2.2.3. Method 3

For wet weight ratio of the gastrocnemius muscle (WWRG), the wet weight of the bilateral gastrocnemius muscles was measured after being carefully dissociated. WWRG was calculated using the ipsilateral/contralateral ratio.

### 2.3. Isolation of Exosomes

The exosomes were isolated through ultracentrifugation (CS120FNX, HITACHI, Japan).

#### 2.3.1. Serum

The supernatant was harvested after being centrifuged at 10,000 g for 30 min and was passed through a 0.22 *μ*m syringe filter and then spun at 100,000 g for 70 minutes. The pellet was resuspended in cold PBS (0.1 M, pH 7.4) after discarding the supernatant and was ultracentrifuged at 100,000 g for 70 min again; then, the pellet (exosomes) was resuspended in 100 *μ*l of PBS and stored in aliquots at -80°C.

#### 2.3.2. Cell Culture Supernatant

The supernatant was centrifuged at 300 g for 10 min to remove cell components and then at 2,000 g for 10 min to remove dead cells. After being centrifuged at 10,000 g for 30 min to eliminate cell debris, the supernatant was subjected to ultracentrifugation for 70 min at 100,000 g. The pellet was resuspended in cold PBS and ultracentrifuged again at 100,000 g for 70 min. Then, the pellet containing exosomes was resuspended in PBS and stored at -80°C.

All operations were performed at 4°C. Exosome concentration was quantified by conducting a Bradford assay (Thermo Fisher Scientific, Colorado, USA).

### 2.4. Characterization of Exosomes

#### 2.4.1. Method 1

For transmission electron microscope (TEM), exosomes were placed on copper and treated with phosphotungstic acid for negative staining. A Tecnai G2 Spirit TEM (Thermo Fisher Scientific) was used to observe the morphology of the exosomes.

#### 2.4.2. Method 2

For western blotting (WB) analysis, exosomes were loaded onto 10% SDS-PAGE gel after protein denaturation. 5% skimmed milk powder was used to block nonspecific binding sites after the protein was transferred onto a polyvinylidene fluoride (PVDF; Millipore) membrane. The blots were incubated with the primary antibodies, CD9 (1 : 2000; Abcam ab92726, Cambridge, MA) and CD63 (1 : 1000; Abcam ab108950), overnight at 4°C. After the membrane was washed with Tris-Buffered Saline and Tween 20, it was incubated with appropriate HRP conjugated secondary antibodies at room temperature for 1 h. The blots were visualized using an electrochemiluminescence reagent (Millipore, Bedford, MA, USA).

#### 2.4.3. Method 3

For nanoparticle tracking analysis (NTA), exosome particle size and concentration were measured using nanoparticle tracking analysis (NTA) using ZetaView PMX 110 (Particle Metrix, Meerbusch, Germany) and corresponding software, ZetaView 8.04.02. The isolated exosome samples had appropriately diluted using 1x PBS buffer, and the ZetaView system was calibrated using 110 nm polystyrene particles. The temperature was maintained at around 23-30°C.

### 2.5. Immunofluorescence Staining

The sections were washed with PBS, and the cell membrane was permeabilized using 0.5% Triton X-100. After blocking using normal goat serum, the samples were incubated with an appropriate primary antibody overnight: Neurofilament 200 (NF200; Sigma-Aldrich N4142, MO, USA) diluted at 1 : 80, GFP (Abcam ab1218) diluted at 1 : 1000, Myelin Basic Protein (Abcam ab40390) diluted at 1 : 200, S-100*α* (Abcam ab11428) diluted at 1 : 200, NGF (Abcam ab6199) diluted at 1 : 100, BDNF (Abcam ab108319) diluted at 1 : 500, and glial cell line-derived neurotrophic factor (GDNF; Abcam ab18956) diluted at 1 : 50. The secondary antibodies used were goat anti-rabbit antibody Alexa 555 (Thermo A-21428) diluted at 1 : 800, goat anti-rabbit antibody Alexa 488 (Thermo A-11034), and goat anti-mouse antibody Alexa 488 (Thermo A-11001) diluted at 1 : 800, while DAPI (Sigma-Aldrich) was used to stain the nuclei.

### 2.6. RNA Extraction

#### 2.6.1. Nerve

500 *μ*l of NucleoZOL was added to the sciatic nerve, and the tissue was homogenized using a tissue homogenizer. The lysate was incubated at room temperature for 15 minutes and centrifuged at 12,000 g for 15 min after 200 *μ*l of RNase-free water was added, and the mixture was shaken vigorously for 15 s. Then, 500 *μ*l of the supernatant was collected in a new centrifuge tube, and 200 *μ*l of 75% ethanol was added. The mixture was incubated at room temperature for 10 min and then centrifuged at 12,000 g for 8 min. The precipitate was collected to obtain long-stranded RNA. Then, the supernatant was transferred, and 500 *μ*l of isopropanol was added and then centrifuged at 12000 g for 15 min. Thereafter, the precipitate was collected to obtain short-stranded RNA.

#### 2.6.2. Exosomes

miRNeasy Serum/Plasma Advanced Kit (Qiagen 217184, Hilden, Germany) was used to extract RNA from the exosomes, as described in the manufacturer's instructions.

### 2.7. RT-qPCR

#### 2.7.1. miRNA

First-strand cDNA was synthesized using a miRcute Plus miRNA First-Strand cDNA Kit (Tiangen Biotech KR211, Beijing, China), while qPCR was performed using a miRcute Plus miRNA qPCR Kit (SYBR Green; Tiangen Biotech FP411). The miR-21-5p primer sequence used was GTAGCTTATCAGACTGATGTTGA, and the internal reference U6 primer sequence used was CTCGCTTCGGCAGCACA. The miR-39 primer was used as external reference (Qiagen). The amplification reaction conditions were as follows: 15 min at 95°C, followed by 40 cycles of denaturation at 94°C for 20 s, followed by the annealing and extension of the primers at 60°C for 34 s. The melting curves were constructed, and each reaction was performed in triplicate. The RT-qPCR data were analyzed using the *ΔΔ*CT method.

#### 2.7.2. mRNA

First-strand cDNA was generated using a FastKing gDNA Dispelling RT SuperMix (Tiangen Biotech KR118), while qPCR was performed using SuperReal PreMix Plus (SYBR Green; Tiangen Biotech FP205). The primer sequences used were as follows: sprouty homolog 2 (SPRY2): TGAAAGACTCCACGGTCTGC, CAGTGAGACTGGCTGCAAGA; Growth-associated protein-43 (GAP43): GAGGGAGATGGCTCTGCTACT, GCTTCATCTACAGCTTCTTTC; DNA Methyltransferase 3A (DNMT3A): CAGCAAAGTGAGGACCATTA, AACACCCTTTCCATTTCAG; and the internal reference, glyceraldehyde-3-phosphate dehydrogenase (GAPDH): ATGACTCTACCCACGGCAAG, GGAAGATGGTGATGGGTTTC. All primer sequences were synthesized by Sangon Biotech. The reaction conditions were as follows: 15 min at 95°C, followed by 40 cycles of denaturation at 95°C for 10 s, annealing of primers at 52°C for 30 s, and extension at 72°C for 32 s. The melting curves were created, and each reaction was performed in triplicate. The data were analyzed using the *ΔΔ*CT method.

### 2.8. Cell Culture

As a rat SC lineage, RSC96 cells purchased from Chinese Academy of Sciences were cultured in Dulbecco's Modified Eagle Medium (DMEM; Corning Cellgro) containing 10% fetal bovine serum (FBS; Gibco). For cell passage, the RSC96 cells were washed with PBS and treated with 0.25% trypsin (Corning Cellgro, Manassas, VA) to digest the cells. Then, the pellet was resuspended in fresh medium after being centrifuged at 1000 g for 5 min.

NG108-15 cells (ATCC, Manassas, VA) were cultured in DMEM supplemented with 10% FBS, 0.1 mM hypoxanthine (Sigma-Aldrich), 400 nM aminopterin (Sigma-Aldrich), and 16 *μ*M thymidine (Sigma-Aldrich) to be used as neutron cell lineage. The protocol for cell passage was the same as that for RSC96, which is mentioned above.

### 2.9. Cell Transfection and Exosome Internalization Assays

#### 2.9.1. Method 1

RSC96 cells were seeded on 0.4 *μ*m Boyden chamber inserts (Corning) which were placed in 24-well plates (Corning). pCT-CD63-GFP plasmids were transfected into RSC96 cells using a Lipofectamine 3000 (Thermo) system after 24 h. The medium was changed 24 h after transfection, and the NG108-15 cells were seeded onto the bottom of a 24-well plate. After 3 days of cocultivation, the NG108-15 cells were fixed using 4% DEPC-PFA and immunofluorescence, and fluorescent in situ hybridization (IF-FISH) was performed.

#### 2.9.2. Method 2

Exosome-free FBS was obtained through ultracentrifugation at 90,000 g for 16 h at 4°C to remove bovine exosomes. Then, the identity of the exosome-free FBS was verified using WB detection of the exosomal markers, CD9 and CD63. The RSC96 cells were cultured in DMEM containing 10% exosome-free FBS for 72 h, and the cell culture supernatant was collected to isolate the exosomes, as mentioned above. NG108-15 cells were seeded onto Nunc™ Glass Bottom Dishes (Thermo), and 96-well plate with DMEM containing 10% exosome-free FBS was added. After 24 h, the cells were divided into 4 groups, as follows. For negative control group (NC), 200 nM miR-21 inhibitor negative control (RiboBio Guangzhou, China) was transfected into NG108-15 cells using a Lipofectamine 3000 system. For negative control + exosomes group (NC + EXO), 200 nM miR-21 inhibitor negative control was transfected into NG108-15 cells, and 3 *μ*g or 0.3 *μ*g/well SC-derived exosomes were added into NG108-15 cells cultured on Nunc Glass Bottom Dishes or 96-well plates, respectively. For miR-21 inhibitor group (IN), NG108-15 cells were transfected with 200 nM miR-21 inhibitors (RiboBio). For miR-21 inhibitor + exosomes group (IN+EXO), NG108-15 cells were transfected with 200 nM miR-21 inhibitors and 3 *μ*g or 0.3 *μ*g/well of SC-derived exosomes, which were added to the NG108-15 cells cultured on Nunc Glass Bottom Dishes or 96-well plates, respectively. Immunofluorescence staining of *β*III-tubulin (1 : 200; Abcam ab18207) and CCK-8 (Dojido, Kumamoto, Japan) was performed after 24 h. Immunofluorescence staining was performed, as mentioned above. For the CCK-8 assays, a 96-well plate was placed in the cell incubator for 2 h after 10 *μ*l of CCK-8 working solution was added to each well. Then, the 450 nm OD value of each well was measured using a microplate reader (Synergy 2, BioTek, United States).

#### 2.9.3. Method 3

RSC96 cells were cultured and divided into 2 groups. For SC-derived exosomes group (EXO-MC), RSC96 cells were infected with the lentivirus produced by the empty plasmid (OBiO Technology). For SC-derived exosomes with the overexpression of miR-21 (EXO-miR-21), RSC96 cells were infected with the lentivirus produced using the miR-21 overexpression plasmid (OBiO Technology). Puromycin-resistant clones were selected by culturing transfected cells for 2 weeks under selective conditions to obtain a stable cell line that overexpressed MC or miR-21. After the two types of cells were cultured in DMEM containing 10% exosome-free FBS for 72 h, the cell culture supernatant was collected, and the exosomes were isolated, as mentioned above.

### 2.10. Immunofluorescence and Fluorescent In Situ Hybridization (IF-FISH)

NG108-15 cells were washed with DEPC-PBS after being fixed using PFA. The following steps were performed, as instructed by the manufacturer (Focobio D-0010, Guangzhou, China). In brief, solutions A and B were added to the cells and allowed to incubate for 20 min and 15 min, respectively. After washing, a DEPC-PBS, hybridization buffer was added for prehybridization, and the mixture was left for 2 h at 55°C. During this time, the hybridization buffer was used to prepare 4 different probes that directly carried Cy3 fluorophore (Sangon Biotech, Shanghai, China): 30 nM U6 probe (positive control), 1200 nM miR-21 probe (target gene), 1200 nM scrambled probe (negative control), and hybridization buffer without probe (blank control). The sequences of the probes are as follows: TCAACATCAGTCTGATAAGCTA (miR-21), CACGAATTTGCGTGTCATCCTT (U6), and GTGTAACACGTCTATACGCCCA (scrambled). These probes were denatured at 85°C for 3 min and equilibrated at 37°C for 2 minutes. Each probe was added and hybridized at 37°C for 24 h after prehybridization. After being washed using a 1 × washing buffer, immunofluorescence double staining of *β*III-tubulin (1 : 200) and GFP (1 : 1000) was performed, as described above, using the corresponding secondary antibodies, donkey anti-rabbit antibody Alexa Fluor 350 (Thermo A-10039) and goat anti-mouse antibody Alexa 488.

## 3. Statistical Methods

All data are presented as the mean ± standard deviation, and statistical analyses were performed using the SPSS 22.0 software (SPSS, Chicago, USA). One-way analysis of variance (ANOVA) with Scheffe's post hoc multiple-comparison analysis was used to analyze intergroup differences, and a *P* value of <0.05 was considered to indicate statistical significance.

## 4. Results

### 4.1. Dual-Directional Regulation of EA on Serum Exosomal miR-21

**Figure 1:**
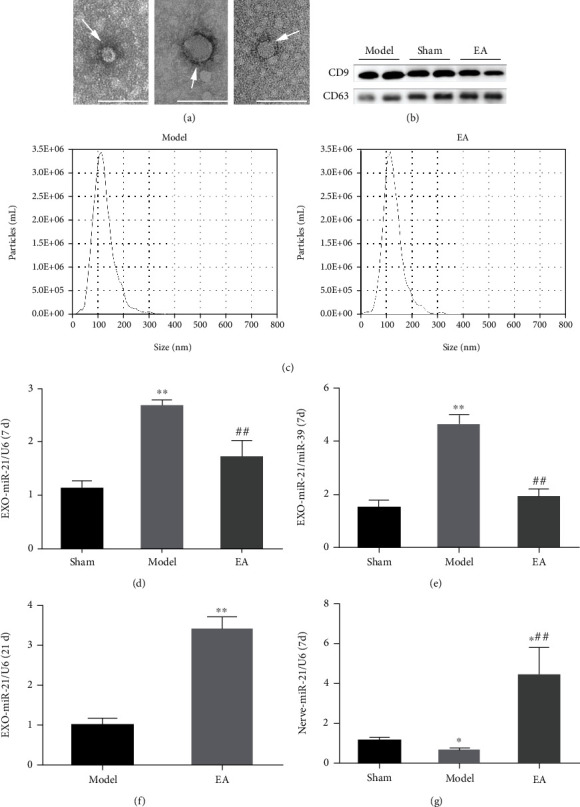
EA regulated the release of serum exosomal miR-21. (a) Exosomes (arrows) were observed under a transmission electron microscope, bar = 100 nm. (b) The surface markers of the exosomes, including CD9 and CD63, were detected using WB. (c) The particle size was detected using NTA. RT-qPCR was conducted to detect the relative expression level of miR-21 in the serum exosomes of rats in each group at 7 d with U6 used as the internal reference (d) or miR-39 used as an external reference (e). ^∗∗^*P* < 0.01 compared with the sham group; ##*P* < 0.01 compared with the model group. (f) RT-qPCR detection of miR-21 expression in serum exosomes at 21 d. ^∗∗^*P* < 0.01 compared with the model group. (g) RT-qPCR detection of the relative expression of miR-21 in locally injured nerves of rats at 7 d. ^∗^*P* < 0.05 compared with the sham group; ##*P* < 0.01 compared with the model group.

**Figure 2:**
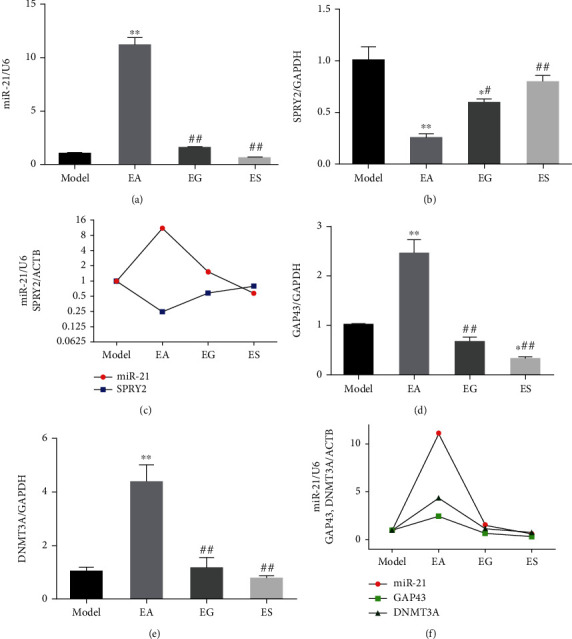
miR-21 regulated gene expression in the distal sciatic nerve. The expression of miR-21 (a) and its target genes, SPRY2 (b), GAP43 (d), and DNMT3A (e), in the injured local nerve were detected using RT-qPCR. ^∗^*P* < 0.05; ^∗∗^*P* < 0.01 compared with the model group; #*P* < 0.05; ##*P* < 0.01 compared with the EA group. The relationship between miR-21 and SPRY2 (c) as well as GAP43 and DNMT3A (f) was summarized.

To investigate the possible involvement of exosomal miR-21 in EA treatment of PNI, a sciatic nerve transection model was constructed (Supplementary Figure S1). The ultracentrifugation method was used to isolate exosomes from the serum at 7 d post-EA treatment. TEM demonstrated that the exosomes were cup-shaped (Figure 1(a)), and WB showed that the expressions of the exosome surface proteins, CD9 and CD63, were positive (Figure 1(b)). To confirm the success of exosome isolation, particle size was detected using NTA. Most exosomes showed a particle size of about 110-120 nm (Figure 1(c)). RT-qPCR was used to test the expression of miR-21 in exosomes. The expression level was significantly elevated in the model compared with the sham group, while its downregulation was observed after EA treatment, compared with the model group (Figure 1(d)). As U6 has not been fully established as an internal reference for exosomal RNA, the external reference miR-39 was used to further verify its expression. It was demonstrated that the expression of miR-21 showed a similar trend using both methods (Figure 1(e)). Interestingly, we found that the level of miR-21 was elevated after EA treatment, compared with the model group, at 21 d (Figure 1(f)). In the damaged local nerves, miR-21 expression was lower in the model group and higher in the EA group, compared with that of the sham group. In addition, EA treatment significantly elevated miR-21 expression, compared with the model group, at 7 d (Figure 1(g)) and 21 d (Figure 2(a)). Taken together, these data show that EA can not only regulate the expression of miR-21 in local injury but also exert a dual-directional regulation effect on serum exosomal miR-21 expression.

### 4.2. Exosomal miR-21 Participated in EA by Promoting the Recovery of Nerve Function and Regeneration

**Figure 3:**
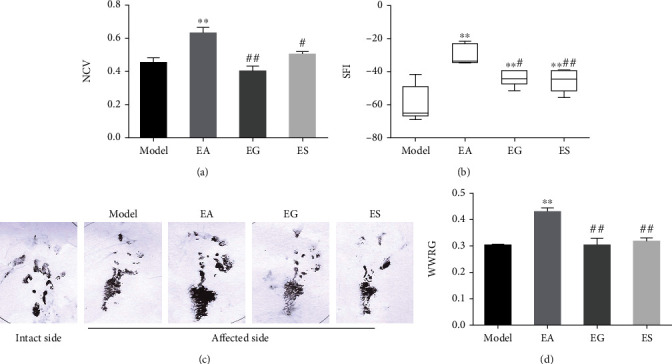
Exosomal miR-21 participates in EA by promoting the recovery of nerve function. NCV (affected side/intact side, (a)), SFI (b, c), and WWRG (affected side/intact side, (d)) were detected in each group. ^∗∗^*P* < 0.01 compared with the model group; #*P* < 0.05; ##*P* < 0.01 compared with the EA group.

**Figure 4:**
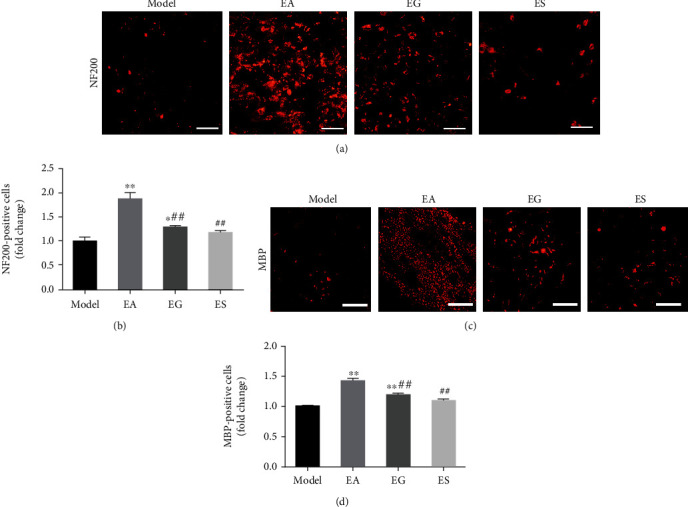
Exosomal miR-21 participates in EA by promoting regeneration of nerve fibers. (a) Immunofluorescence staining of NF200 (red). Bar = 50 *μ*m. (b) The number of NF200 positive cells indicates the number of regenerated axons. ^∗^*P* < 0.05; ^∗∗^*P* < 0.01 compared with the model group; ##*P* < 0.01 compared with the EA group. (c) Immunofluorescence staining of MBP (red). Bar = 25 *μ*m. (d) Statistics of the number of MBP positive cells, which indicated the number of myelin sheaths. ^∗∗^*P* < 0.01 compared with the model group; ##*P* < 0.01 compared with the EA group.

GW4869 and miR-21-5p-sponge-lentivirus were used to explore whether exosomal miR-21 is involved in the effect produced by EA. The expression of miR-21 in local injured tissue increased in the EA group, compared with that of the model group, and decreased in the EG and ES groups, compared with that of the EA group at 21 d (Figure 2(a)). The expression level of SPRY2, a target gene of miR-21, was opposite to the expression of miR-21 (Figures 2(b) and 2(c)). Moreover, the expression levels of GAP43 and DNMT3A, which are positively correlated with axon growth, were positively correlated with miR-21 expression (Figures 2(d)–2(f)). Severe plantar ulcers were observed to have appeared in the EG and ES group at 21 d. In the EA group, there was no total absence, and no large-area ulcers were observed on the plantar, while the ulcer surface healed well without exudation (Supplementary Figure S2). Compared with the model group, EA treatment significantly increased NCV (Figure 3(a)), SFI (Figures 3(b) and 3(c)), and WWRG (Figure 3(d)) after 21 d, which indicated a higher sciatic nerve function recovery index. Additionally, the values of these 3 indicators were significantly downregulated in the EG and ES group, compared with the EA group. Immunofluorescence staining of NF200 and MBP was used to detect the regeneration of axons and myelin sheath of the distal damaged tissue. The number of regenerated axons and myelin sheaths showed that EA is a powerful tool that can be used to promote the regeneration of nerve fibers, compared with the model group, while the regeneration of the nerve fiber was significantly inhibited in the EG and ES group, compared with the EA group (Figure 4).

### 4.3. Exosomal miR-21 Participates in EA by Promoting the Proliferation of SC and the Expression of NTFs

**Figure 5:**
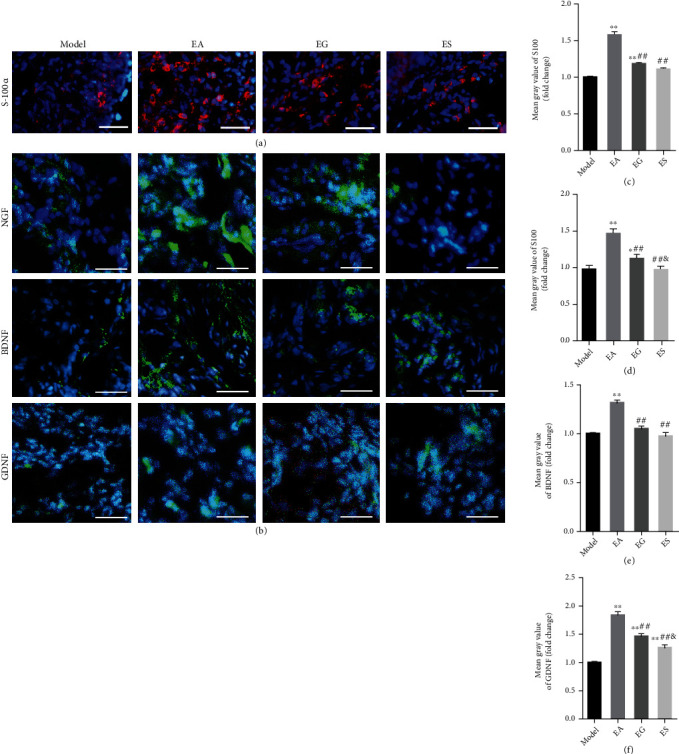
Exosomal miR-21 participates in EA by promoting the proliferation of SC and the expression of NTFs. (a) Immunofluorescence staining of S-100*α* (red). The nuclei were visualized using DAPI (blue). Bar = 50 *μ*m. (b) Immunofluorescence staining of NGF, BDNF, and GDNF (green). Bar = 20 *μ*m. (c) Mean fluorescence intensity of S-100*α*. ^∗∗^*P* < 0.01 compared with the model group; ##*P* < 0.01 compared with the EA group. Mean fluorescence intensity of NGF, BDNF, and GDNF (d–f). ^∗^*P* < 0.05; ^∗∗^*P* < 0.01 compared with the model group; ##*P* < 0.01 compared with the EA group; &*P* < 0.05 compared with the EG group.

S-100*α* is found in the SC cytoplasm and myelin sheath of peripheral nerves and functions as an objective morphological indicator that reflects the proliferation of SC *in vivo*. Nerve regeneration was indirectly evaluated through the proliferation of SC and the expression of NTFs (NGF, BDNF, GDNF). We observed that S-100*α*, NGF, BDNF, and GDNF expression levels were upregulated in the EA group, compared with that of the model group, while these expression levels were inhibited in the EG and ES group, compared with the EA group according to immunofluorescence staining (Figure 5).

### 4.4. Exosomal miR-21 Regulates Gene Expression Changes in the Distal Sciatic Nerve

**Figure 6:**
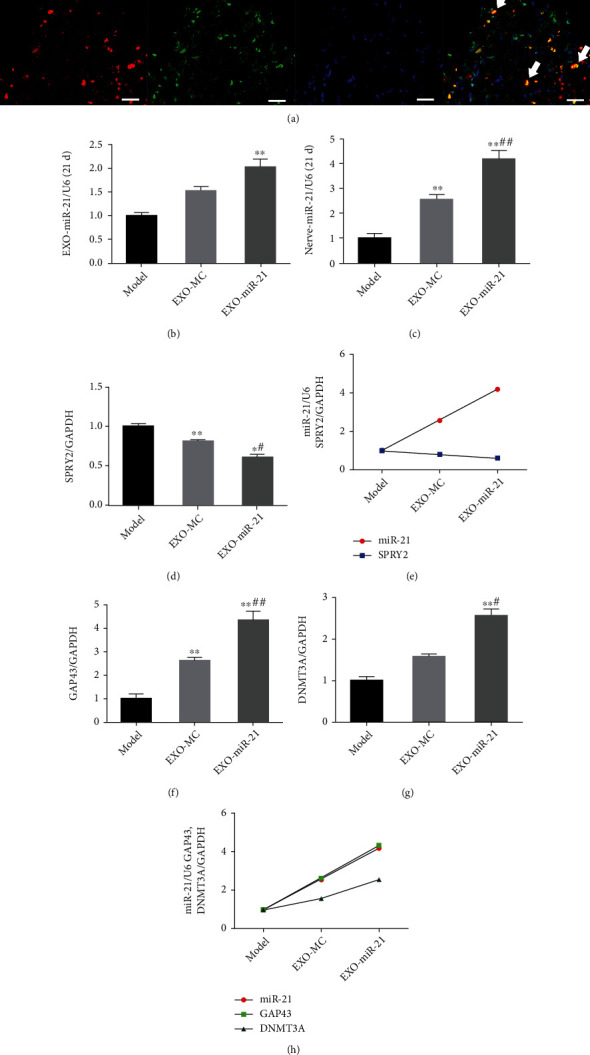
Exosomal miR-21 regulates the gene expression levels in the distal sciatic nerve. (a) Immunofluorescence staining of NF200 (red) and GFP (green). The nuclei were visualized using DAPI (blue). The colocalization of axons and exosomes was marked with arrows. Bar = 25 *μ*m. (b) Relative expression level of miR-21 in serum exosomes at 21 d. (c) Relative expression of miR-21 in injured tissue at 21 d. Relative expression level of SPRY2 (d), GAP43 (f), and DNMT3A (g). ^∗^*P* < 0.05; ^∗∗^*P* < 0.01 compared with the model group; #*P* < 0.05; ##*P* < 0.01 compared with the EXO-MC group. The relationship between miR-21 and SPRY2 (c) or that of GAP43 and DNMT3A (f) was summarized.

Exosomes secreted from local SC were labeled using GFP through lentiviral transfection. Colocalization of NF200 and GFP can be used as evidence of the secretion of exosomes by SC can be transferred and internalized to axons (Figure 6(a)). Next, we isolated exosomes that overexpressed miR-21 or normal exosomes from the cell supernatant of SC with or without lentiviral infection. After PCR verification (Supplementary Figure S3), these exosomes were injected into the injury region of the model. At 21 d, we found that the expression of miR-21 was elevated in serum exosomes and injured tissues in the EXO-MC and EXO-miR-21 groups, compared with that of the model group, and the increase in the EXO-miR-21 group was more pronounced than that of the EXO-MC group (Figures 6(b) and 6(c)). The expression of SPRY2 was the lowest in the EXO-miR-21 group, and the trend of SPRY2 expression showed a negative correlation with miR-21 (Figures 6(d) and 6(e)). Injection of exosomes that overexpressed miR-21 significantly enhanced the expression level of GAP43 and DNMT3A (Figures 6(f) and 6(g)). The expression of GAP43 and DNMT3A showed a positive correlation with miR-21 (Figure 6(h)).

### 4.5. Exosomal miR-21 Participates in Nerve Regeneration and Repair

**Figure 7:**
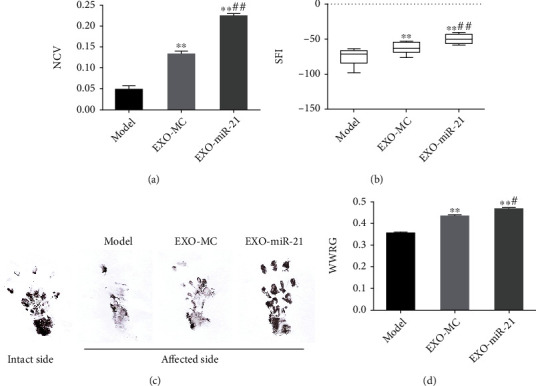
Exosomal miR-21 participates in the recovery of nerve function. NCV (affected side/intact side, (a)), SFI (b, c), and WWRG (affected side/intact side, (d)) were detected in each group. ^∗∗^*P* < 0.01 compared with the model group; #*P* < 0.05; ##*P* < 0.01 compared with the EXO-MC group.

**Figure 8:**
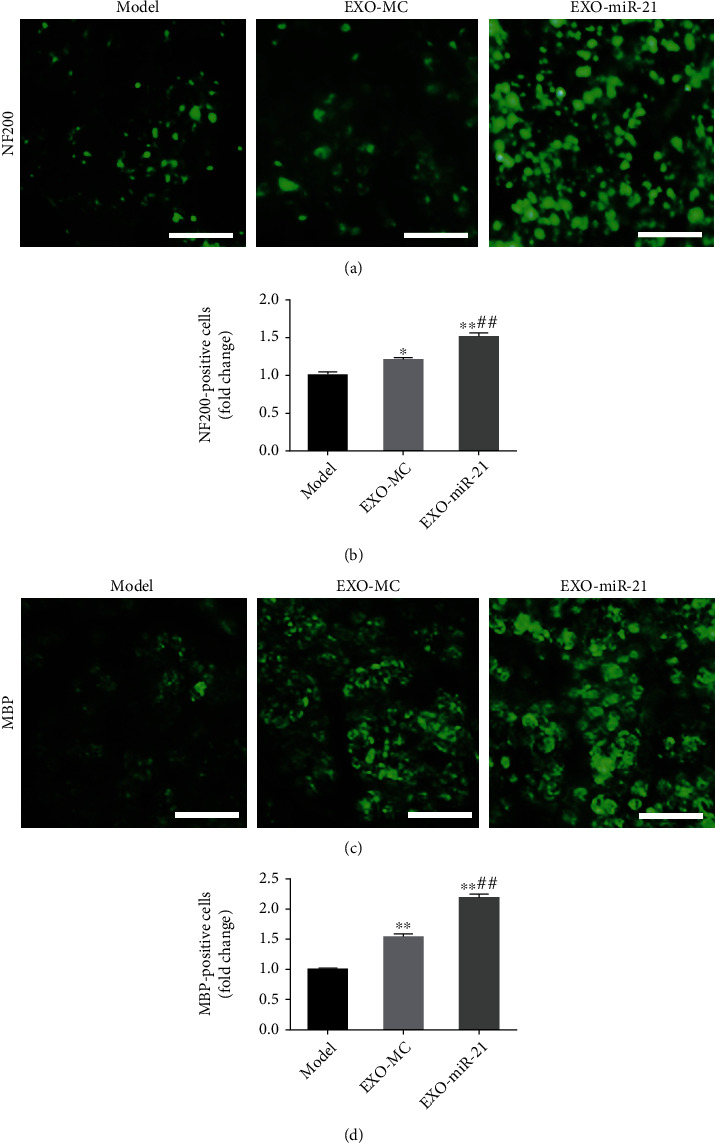
Exosomal miR-21 participates in the regeneration of nerve fibers. (a) Immunofluorescence staining of NF200 (green). Bar = 10 *μ*m. (b) The number of NF200 positive cells, which was used to indicate the number of regenerated axons, was counted. ^∗^*P* < 0.05; ^∗∗^*P* < 0.01 compared with the model group; ##*P* < 0.01 compared with the EXO-MC group. (c) Immunofluorescence staining of MBP (green). Bar = 10 *μ*m. (d) Statistics of the number of MBP positive cells, which was used to indicate the number of myelin sheaths. ^∗∗^*P* < 0.01 compared with the model group; ##*P* < 0.01 compared with the EXO-MC group.

Symptoms of plantar ulcers were alleviated in the EXO-MC and EXO-miR-21 group, compared with that of the model group (Supplementary Figure S4). The sciatic nerve function recovery index improved after 21 d of EXO-MC and EXO-miR-21 treatment, as indicated through changes in NCV, SFI, and WWRG. In addition, NCV, SFI, and WWRG values in the EXO-miR-21 group were dramatically higher than those in the EXO-MC group (Figures 7(a)–7(d)). Immunofluorescence staining of NF200 and MBP indicated that EXO-MC and EXO-miR-21 were involved in promoting the regeneration of nerve fibers, compared with the model group. Furthermore, the number of NF200 and MBP positive cells was greater in the EXO-miR-21 group, compared to the EXO-MC group (Figures 8(a)–8(d)).

### 4.6. Exosomal miR-21 Participates in the Proliferation of SC and the Expression of NTFs

**Figure 9:**
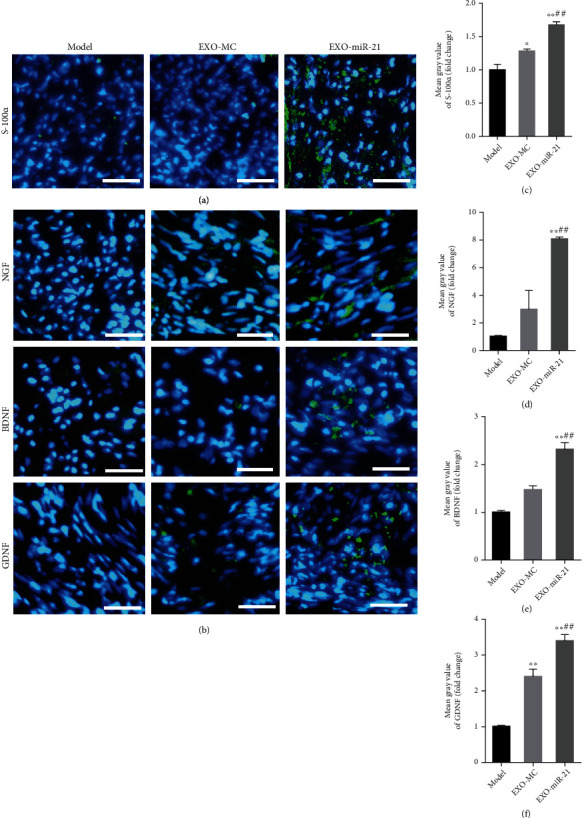
Exosomal miR-21 participates in the proliferation of SC and the expression of NTFs. (a) Immunofluorescence staining of S-100*α* (green). The nuclei were visualized using DAPI (blue) staining. Bar = 10 *μ*m. (b) Immunofluorescence staining of NGF, BDNF, and GDNF (green). Bar = 10 *μ*m. (c) Mean fluorescence intensity of S-100*α*. ^∗^*P* < 0.05; ^∗∗^*P* < 0.01 compared with the model group; ##*P* < 0.01 compared with the EXO-MC group. Mean fluorescence intensity of NGF, BDNF, and GDNF (d–f). ^∗∗^*P* < 0.01 compared with the model group; ##*P* < 0.01 compared with the EXO-MC group.

Since SC proliferation and NTF secretions play a key role in peripheral nerve regeneration, we detected the proliferation of SC and the expression of NTFs using immunofluorescence staining. EXO-MC treatment significantly promoted the expression of S-100*α*, which indicated that EXO-MC is involved in the proliferation of SC after PNI. There were more S-100*α* positive cells in the injured nerve of rats treated using EXO-miR-21, compared with the EXO-MC group. Although the expressions of NGF, BDNF, and GDNF were upregulated in the EXO-MC group, levels were much higher in the EXO-miR-21 group, demonstrating that EXO-miR-21 injection not only further enhanced the proliferation of SC but also promoted levels of NTFs, including NGF, BDNF, and GDNF probably by transferring much more miR-21 (Figure 9).

### 4.7. SC-Derived Exosomal miR-21 Promotes Neurite Outgrowth In Vitro

**Figure 10:**
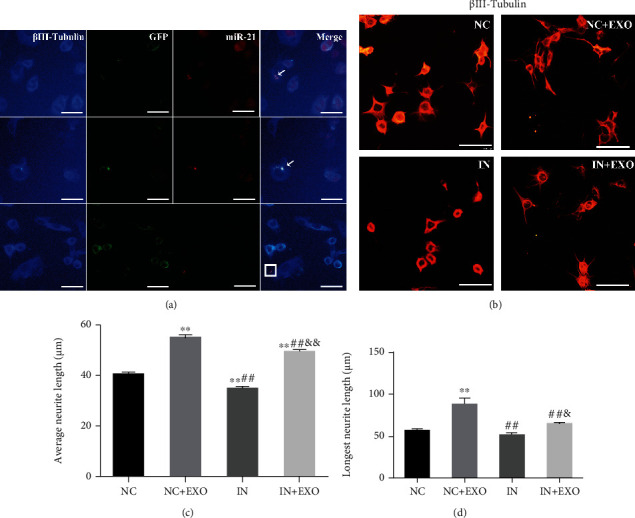
SC-derived exosomal miR-21 promotes neurite outgrowth *in vitro*. (a) miR-21 in situ hybridizations (red) and immunofluorescence staining of *β*III-tubulin (blue) showed that exosomes secreted by SC could be taken up by neurons. miR-21 was found in the neuron cell body (indicated by arrows) and neuronal processes (shown in the square). Bar = 25 *μ*m. (b) Immunofluorescence staining of *β*III-tubulin (red) to detect NG108-15 protrusion growth. Bar = 50 *μ*m. (c) The average protrusion length of the neurons were calculated. ^∗∗^*P* < 0.01 compared with the NC group; ##*P* < 0.01 compared with the NC + EXO group; &&*P* < 0.01 compared with the IN group. (d) The length of the longest protrusion was calculated. ^∗∗^*P* < 0.01 compared with the NC group; ##*P* < 0.01 compared with the NC + EXO group; &*P* < 0.05, compared with the IN group.

The exosomes secreted by SC were labeled using GFP and were cocultivated with NG108-15 cells for 3 d. Double-immunofluorescence staining indicated that the exosomes secreted by SC could be taken up by neurons (Supplementary Figure S5). Moreover, we found that SC-derived exosomal miR-21 could be taken up by neurons and their neurites using IF-FISH (Figure 10(a)). FBS without exosomes (FBS-EXO) were harvested after the exosomes were successfully removed (Supplementary Figure S6A) and were found to be positive for CD9 and CD63 (Supplementary Figure S6B). Neurons were treated with SC-derived exosomes, and the average and longest length of the neurites were increased in the NC + EXO group, compared with the NC group. The growth of neurites was attenuated when the expression of miR-21 was inhibited. Furthermore, the effects of EXO treatment were inhibited in the IN+EXO group, compared with the NC + EXO group (Figures 10(b)–10(d)). Taken together, SC-derived exosomal miR-21 promoted neurite outgrowth *in vitro.* In addition, the viability of NG108-15 cells in each group was detected using CCK8 assay, and viability of cells in the IN+EXO group was much lower than that of the NC + EXO group (Supplementary Figure S7).

## 5. Discussion

Although peripheral nerves have a reconstitutive ability, it is difficult to fully recover nerve function after PNI. EA is not only effective in treating PNI [53] but can also be used for the treatment of neuropathic pain [54] and central nervous disease [55]. Since miRNAs are closely associated with the development of PNI [15, 32, 33, 41, 56–59] and nerve repair, while exosomes are valuable carriers of miRNAs [60], our study focused on exosomal delivery of miR-21 to explore the effects of EA. Exosomes can minimize safety concerns associated with the administration of live cells as cell-free therapy and has a great therapeutic potential for the treatment of brain, heart, liver, lung, skin, and bone diseases [61]. Thus far, it has been identified that miR-21-5p contains exosomes derived from SC that can improve sensory neuron growth through the PTEN-PI3K pathway *in vitro* [62] and has the ability to ameliorate peripheral neuropathy in type 2 diabetic mice [63]. In addition, exosomal cargo, including miR-21, can regulate sensory neurons for macrophage communication after nerve trauma [42], indicating that exosomal miR-21 has the potential to mediate and treat PNI. Levels of the exosomal biomarkers, TSG101 and CD81, increased in the ischemic striatum after EA treatment [64], suggesting that EA may stimulate an increase in exosomes secreted. Therefore, the regulation of secretions and transfer of exosomes between cells may be one of the principal mechanisms of EA treatment.

We first explored whether the release of exosomes and the delivery of miR-21 by exosomes in the PNI model rats were affected by EA. The acupoints, “Huantiao” (GB30) and “Zusanli” (ST36), were selected in our study based on basic traditional Chinese medicine theory. “Huantiao” (GB30) is located at the foot of the *Shaoyang* gallbladder meridian and intersects with the foot bladder meridian. EA can invigorate blood in the lower limbs. “Zusanli” (ST36) is located in the foot *Yangming* stomach meridian and perform many functions, such as health care and strongness. PNI belongs to the dysfunction syndrome in Chinese medicine. *Huangdi Neijing* indicates that the treatment of dysfunction is based on *Yangming*. It was found that the expression level of miR-21 in serum exosomes was lower than that of the model group at 1 week after EA treatment but was higher at 3 weeks, while miR-21 expression in injured local nerves in the EA group was consistently elevated, compared with the model group. It showed that EA inhibits the release of serum exosomal miR-21 in PNI rats during the early stage of PNI, while promoting its release during later stages. We speculated that EA may promote the transfer of exosomal miR-21 from serum to injured tissue. Since SC is a unique type of glia cell in the peripheral nervous system and exosomes are the main mediators of glia-neuron communication, the lentivirus of pCT-CD63-GFP was used for green fluorescence labeling of the exosome, which may be secreted by SC and double-immunofluorescence staining showed that these exosomes can be taken up by axons.

Since EA exerts a dual-directional regulatory effect on serum exosomal miR-21, we explored whether exosomal miR-21 was involved in the promotion of nerve regeneration exerted by EA. GW4869 is a noncompetitive sphingomyelinase inhibitor that inhibits the release of exosomes [65, 66]. Intraperitoneal injection of GW4869 can inhibit exosomes in circulation [67], and there were no adverse effects reported following the use of GW4869 [68, 69]. The miR-21-5p-sponge-lentivirus was used to inhibit the expression of miR-21 in the injured nerve tissue, which included exosome-derived miR-21. Based on the sciatic nerve function recovery index and immunofluorescence staining of NF200, MBP, S-100*α*, and NTFs, we proved that EA effectively promoted the recovery of nerve function after PNI. However, the reduction in circulatory exosomal release or miR-21 expression in the local injured tissue impaired the effect of EA. Therefore, the delivery of miR-21 by exosomes may be a key mechanism by which the effect of EA therapy is exerted. Based on this, we further tested whether SC exosomes overexpressing miR-21 were conducive to the recovery of PNI. The exosomes secreted by normal SC showed an obvious ability to promote the recovery of nerve function, and interestingly, injection of exosomes overexpressing miR-21 provided more encouraging outcomes. In addition, exosomal miR-21 secreted by SC could promote neurite outgrowth *in vitro*. Taken together, SC-derived exosomal miR-21 plays a significant role in the promotion of the repair effect exerted by EA, and exosomes may be potential carriers and transporters of miR-21 mediated by EA after PNI.

As an extensively studied miRNA, miR-21 can mediate inflammation [70], oxidative stress [71], cell apoptosis [72], proliferation [73], and regulate multiple signal pathways, including the protein kinase B (AKT) signaling pathway [74, 75], extracellular regulated protein kinases (ERK) signaling pathway [76, 77], and nuclear factor kappa-B (NF-*κ*B) [78]. Plenty of studies have proven that miR-21 participates in the occurrence and development of tumors [79, 80], cardiovascular [81] and lung diseases [82, 83], glaucoma intraocular pressure [84], age-related skin wound healing [85], sepsis, and acute kidney injury [86]. Moreover, it is expected to be a potential marker for the diagnosis of diseases, such as colon cancer [87], liver cancer or hepatitis severity [88], colorectal cancer [89], and lung adenocarcinoma [90]. The expression of miR-21 was significantly elevated at 1, 4, 7, and 14 days after PNI and was able to regulate the gene expression of the sciatic nerve stump [91]. In fact, miR-21 exerted a protective effect on central neuron apoptosis [92]. Moreover, miR-21 could promote the proliferation of SC [43], inhibiting SC apoptosis [93], and enhancing the differentiation of stem cells towards SC [94]. In this study, we found that miR-21 showed a significant negative correlation with its target gene, SPRY2, during PNI and the repair process. miR-21 can promote the repair of nerve fibers by directly targeting SPRY2 [40], and the knockout of SPRY2 can enhance axonal regeneration of DRG neurons [95]. However, its in-depth mechanisms need to be further explored. GAP43 is related to neuronal development and plasticity [96]. It can support long-distance axon growth [97], and its deficiency can cause abnormal neuron pathfinding [98]. DNMT3A plays a significant role in the regulation of neuronal function and is essential for the synaptic plasticity of the central nervous system [99]. A lack of DNMT3A can lead to a reduction of motor neurons, degeneration of the nervous system, and defects of neuromuscular functions [100]. GAP43 [101, 102] and DNMT3A [103] are proteins associated with axon growth. In this study, we found that miR-21 was positively correlated with GAP43 and DNMT3A expression levels, while this potential link remains to be further elucidated.

As an SC cell line, RSC96 has been used in many studies [104–107], and results of experiments conducted on it are consistent with primary SC in common cell experiments [108, 109]. NG108-15 cells have been widely used as neuron cell lines [102, 110–113] to imitate motor neuron-like peripheral neurons to observe the growth of neurites using *β*III-tubulin staining [102] *in vitro*. At present, we use these two cell lines to harvest sufficient exosomes and reduce the difficulty of the use of neuron culture. However, reliability verification of the primary cells awaits further investigation. As a carrier, exosomes protect their contents and transfer their cargo to target cells to exert certain functions [114]. Exosomes can be used to avoid the immune response, cancer risk, and ethical issues, compared with cell therapy [115]. Interestingly, we can transform and modify exosomes and turn them into handier “weapons” for disease treatment. For example, cells can be modified to secrete more exosomes using specific RNA packages [116]. Cell nano-perforation can stimulate cells to produce up to 50 times more functional exosomes, which are equally effective, even in cells with low basal secretion levels [117]. We can also learn from the production and action methods of exosomes to continuously squeeze cells through a nano-sized filter to obtain exosome-mimetic nanovesicles. The resulting output was 100 times higher than that of exosomes produced in a traditional way and exerted the same functions [118, 119]. All these results demonstrate that exosomes have an immense potential as a novel method of treatment for PNI, especially after being modified.

However, this study has certain limitations: miR-21 can regulate many signaling pathways, such as the PI3K/Akt and Wnt/beta-catenin pathways, which are involved in the repair of neuron injury [62]; therefore, the involvement of these signaling pathways should be investigated further. We found that miR-21 expression levels in the location of injury in the exosome group was higher than that of the control group *in vivo* at 21 days postsurgery. However, it needs to be investigated whether sustained elevated levels of miR-21 can still promote the repair of peripheral nerves, and the dosage and administration route of miR-21 needs to be further verified. In addition, it is better to verify these details using experiments conducted on primary cells *in vitro*. Moreover, exosomes exist not only in the SC but also in the sciatic nerve itself and serum, which are the major origins of elevated levels of miR-21 expression in the local injury nerve and are worth exploring. Exosomes can mediate the transmission of many noncoding RNAs and proteins, including miR-21 [50], and the clarification of phenotypic differences and functional specificity of exosomes secreted from SC, serum and axon would be of great significance. At the same time, the specific mechanism by which miR-21 promotes nerve repair, such as the effects of miR-21 on SC differentiation, the guidance of newborn axons, myelin clearance, and the recruitment of macrophages, needs to be elucidated further. Meanwhile, exosomes can be combined with material science to create specific viscus and slow release treatments that would be of immense value for the development of other methods of treatment in the future.

Taken together, our results demonstrated the mechanism by which EA exerts its effects from the perspective of exosomal delivery of miR-21 and provides a theoretical basis for exosomal miR-21 as a novel method of PNI therapy.

## Data Availability

The data that support the findings of this study are available from the corresponding author upon reasonable request.

## Supplementary Materials

Supplementary Materials Supplementary Figure S1: construction of PNI model of rat. (A) The sciatic nerve was dissociated. (B) After cutting the sciatic nerve, the epineurium on both sides of the stumps were sutured. Supplementary Figure S2: the general condition of the affected foot of rats was observed after being treated by EA EG or ES. Supplementary Figure S3: the expression of miR-21 in exosomes was detected by RT-qPCR. ^∗∗^*P* < 0.01, compared with the EXO-MC group. Supplementary Figure S4: the general condition of the affected foot of rats was observed after being treated by exosomes. Supplementary Figure S5: *β*III-Tubulin (red) and GFP (green) showed that exosomes secreted by SC were taken up by NG108-15 cells. Nuclei were visualized with Hoechst 33342 (blue). The upper arrow showed that the SC exosomes entered into the neuron processes, and the lower arrow showed that the SC exosomes were still outside the neuron. Bar = 50 *μ*m. Supplementary Figure S6: exosomal markers CD9 and CD63 were detected by WB. Supplementary Figure S7: CCK-8 detection of cell viability in each group of NG108-15. ^∗∗^*P* < 0.01, compared with the NC group; ##*P* < 0.01, compared with the NC + EXO group; &&*P* < 0.01, compared with the IN group.
