# Supplementary material for: Exosome-Mediated miR-21 Was Involved in the Promotion of Structural and Functional Recovery Effect Produced by Electroacupuncture in Sciatic Nerve Injury
Source: Oxid Med Cell Longev. 2022 Jan 29;2022:7530102. doi: 10.1155/2022/7530102 (PMC8817850; doi:10.1155/2022/7530102)
Supplement: Supplementary Materials — Supplementary Figure S1: construction of PNI model of rat. (A) The sciatic nerve was dissociated. (B) After cutting the sciatic nerve, the epineurium on both sides of the stumps were sutured. Supplementary Figure S2: the general condition of the affected foot of rats was observed after being treated by EA EG or ES. Supplementary Figure S3: the expression of miR-21 in exosomes was detected by RT-qPCR. ∗∗P < 0.01, compared with the EXO-MC group. Supplementary Figure S4: the general condition of the affected foot of rats was observed after being treated by exosomes. Supplementary Figure S5: βIII-Tubulin (red) and GFP (green) showed that exosomes secreted by SC were taken up by NG108-15 cells. Nuclei were visualized with Hoechst 33342 (blue). The upper arrow showed that the SC exosomes entered into the neuron processes, and the lower arrow showed that the SC exosomes were still outside the neuron. Bar = 50 μm. Supplementary Figure S6: exosomal markers CD9 and CD63 were detected by WB. Supplementary Figure S7: CCK-8 detection of cell viability in each group of NG108-15. ∗∗P < 0.01, compared with the NC group; ##P < 0.01, compared with the NC + EXO group; &&P < 0.01, compared with the IN group. [file 7530102.f1.docx]

**Electroacupuncture promotes structural and functional recovery after sciatic nerve injury of rat through regulation of exosome-mediated miR-21**

Supplemental figures:


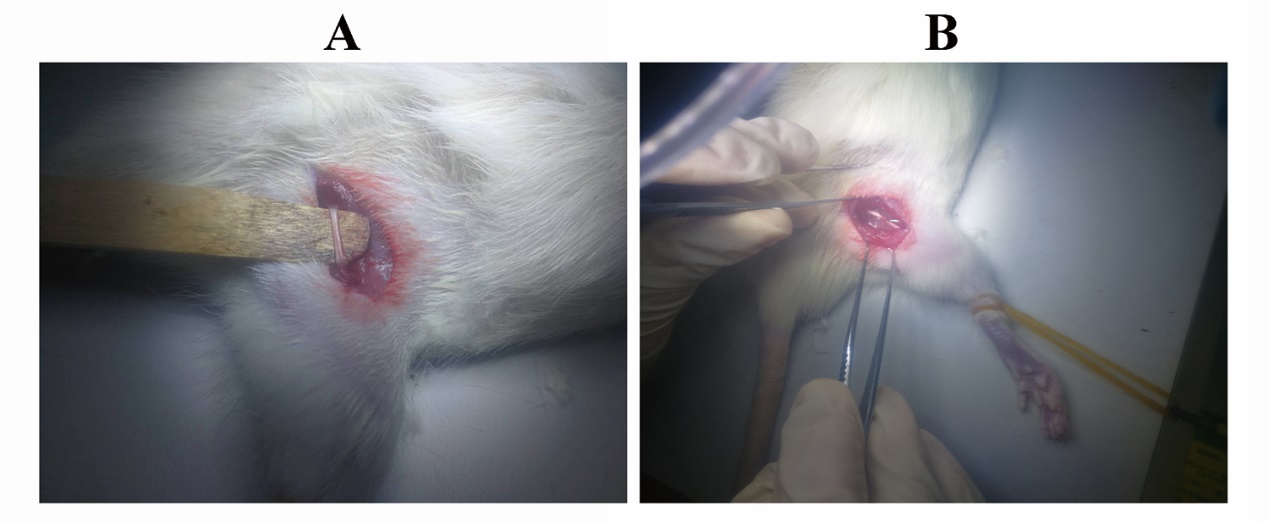


**Supplementary Figure S1.** Construction of PNI model of rat. (A) The sciatic nerve was dissociated. (B) After cutting the sciatic nerve, the epineurium on both sides of the stumps were sutured.


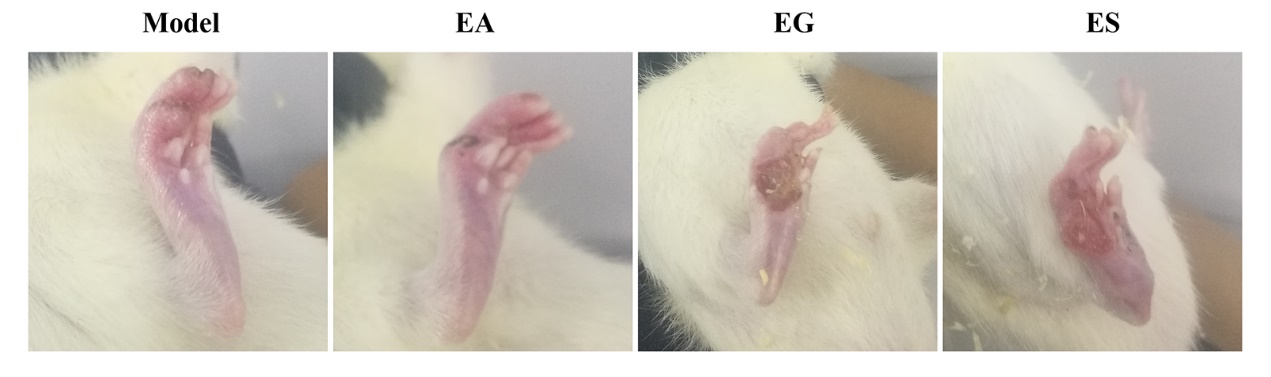


**Supplementary Figure S2.** The general condition of the affected foot of rats was observed after being treated by EA EG or ES.





**Supplementary Figure S3.** The expression of miR-21 in exosomes was detected by RT-qPCR. ***P*<0.01, compared with EXO-MC group.


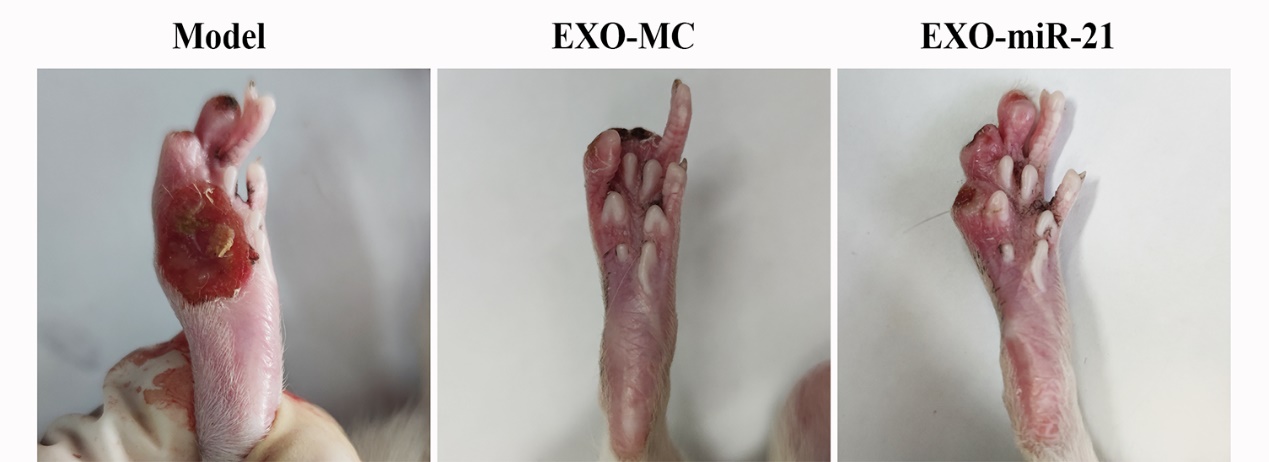


**Supplementary Figure S4.** The general condition of the affected foot of rats was observed after being treated by exosomes.


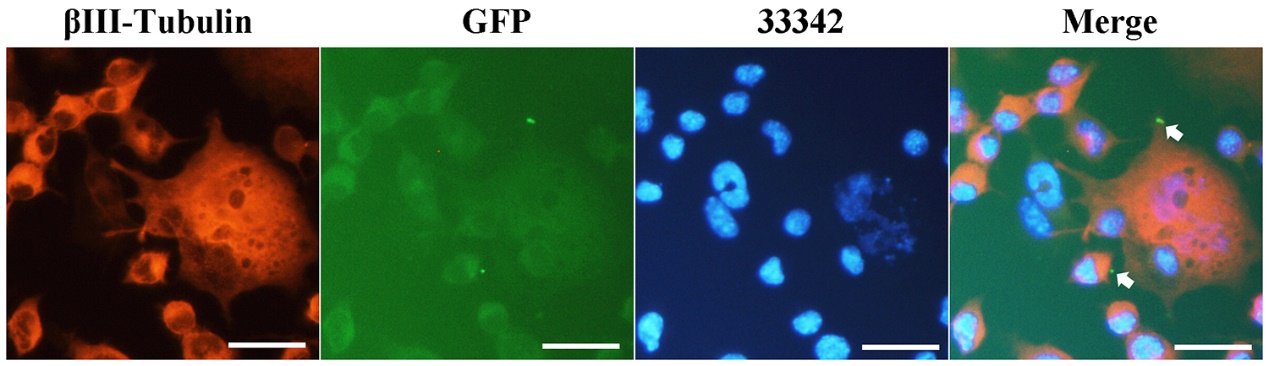


**Supplementary Figure S5.** βIII-Tubulin (red) and GFP (green) showed that exosomes secreted by SC were taken up by NG108-15 cells. Nucleus were visualized with Hoechst 33342 (blue). The upper arrow showed that the SC exosomes entered into the neuron processes, and the lower arrow showed that the SC exosomes were still outside the neuron. Bar = 50 μm.


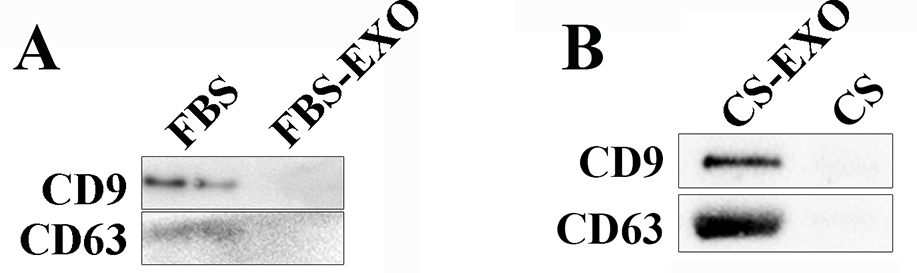


**Supplementary Figure S6.** Exosomal markers CD9 and CD63 were detected by WB.





**Supplementary Figure S7.** CCK-8 detection of cell viability in each group of NG108-15. ***P*<0.01, compared with NC group; ##*P*<0.01, compared with NC+EXO group; &&*P*<0.01, compared with IN group.
